# Case report: Successful perioperative intervention with efgartigimod in a patient in myasthenic crisis

**DOI:** 10.3389/fimmu.2025.1524200

**Published:** 2025-01-28

**Authors:** Shahar Shelly

**Affiliations:** Department of Neurology, Rambam Medical Center, Haifa, Israel

**Keywords:** myasthenia gravis, efgartigimod, myasthenic crisis, very-late-onset myasthenia gravis, case report

## Abstract

**Introduction:**

This case describes successful response to efgartigimod in the treatment of myasthenic crisis secondary to paraneoplastic disease, and in the perioperative setting.

**Methods:**

An elderly female presented with speech difficulties, cessation of eating and 10kg weight loss over 4 months.

**Results:**

Examination revealed ptosis, dysarthria, nasal speech, and weakness in limbs and neck flexors. Single fiber electromyography demonstrated abnormal jitter response in the orbicularis oculi muscle. Nicotinic acetylcholine receptor antibodies were detected in serum. The patient was diagnosed with very-late-onset myasthenia gravis (MG) in a myasthenic crisis and later required intubation and admission to intensive care but was unresponsive to plasma exchange. Paraneoplastic disease was suspected and computed tomography revealed a bladder mass. Efgartigimod 10 mg/kg was administered intravenously to stabilize her condition before surgery. The patient’s Myasthenia Gravis Activities of Daily Living (MG-ADL) score decreased from 19 to 14 after the first dose and she subsequently underwent surgical removal of the bladder tumor without complication. Her condition continued to improve post-operatively with completion of the first treatment cycle. Four cycles of efgartigimod over 10 months resulted in an MG-ADL score of 3.

**Discussion:**

Efgartigimod may be a novel treatment for perioperative management of MG, myasthenic crisis, and paraneoplastic MG. Further study is warranted.

## 1 Introduction

Paraneoplastic myasthenia gravis (MG) is characterized by non-limb symptoms such as bulbar, ocular, neck, and respiratory symptoms, and a rapid progression to severe disability (1, 2). While often associated with thymoma, extrathymic malignancies have also been reported in paraneoplastic MG (3). Surgical removal of the underlying neoplasm is a critical step in disease management (1); however, the physiological stress response induced by surgery poses a significant challenge in patients with unstable MG, as it can potentially exacerbate myasthenic symptoms and lead to complications (4, 5).

Myasthenia gravis is an autoimmune disorder caused by antibody-mediated disruption of neuromuscular transmission, primarily due to autoantibodies targeting the acetylcholine receptor (AChR) at the neuromuscular junction. This results in impaired synaptic transmission, leading to fluctuating muscle weakness and fatigue (1). In cases of myasthenic crisis, respiratory and bulbar dysfunction can rapidly escalate, necessitating urgent intervention (6). Short-term therapies, such as plasma exchange (PLEX) and intravenous immunoglobulin (IVIg), are standard treatments for managing myasthenic crisis, particularly in cases involving significant respiratory or bulbar dysfunction (1, 6, 7). PLEX works by removing circulating autoantibodies, providing a relatively rapid therapeutic effect, while IVIg modulates immune response and reduces autoantibody levels. Both treatments are also commonly employed for preoperative stabilization to minimize perioperative exacerbation of MG symptoms. However, therapeutic responses to these conventional treatments are not universally consistent, especially in complex or refractory cases.

These interventions are also commonly utilized for preoperative stabilization to minimize perioperative exacerbation of MG symptoms. However, therapeutic responses to these conventional treatments are not universally consistent, especially in complex or refractory cases. This report presents the perioperative use of efgartigimod, a neonatal Fc receptor (FcRn) antagonist, in an elderly patient with myasthenic crisis secondary to active paraneoplastic disease who was unresponsive to conventional therapies. The case emphasizes the potential role of efgartigimod as an emerging therapeutic option, enabling both preoperative stabilization and sustained postoperative improvement in a challenging clinical scenario.

### 1.1 Case report

An 86-year-old female presented to the emergency room with a 4-month history of speech difficulties, cessation of eating and weight loss of 10kg. Her past medical history was significant for hypertension and cataracts. On neurological examination she had right-sided ptosis covering half the iris, severe dysarthria, nasal speech, proximal weakness in upper and lower limbs, and weakness of neck flexors. An electrocardiogram revealed normal sinus rhythm, while laboratory findings indicated leukocytosis (11,000 cells/µL), hypokalemia (3.1 mmol/L) and a hemoglobin level of 11.7 g/dL. Computed tomography (CT) of the brain showed old established lacunar infarcts in the basal ganglia. A troponin test revealed elevated levels, which coupled with her neurological symptoms, and in the absence of any significant cardiac findings, indicated a neuromuscular junction disorder. She underwent single fiber electromyography which demonstrated abnormal jitter response in the left orbicularis oculi muscle, and she tested positive for nicotinic acetylcholine receptor (AChR) antibodies. She was diagnosed with very-late-onset MG in a myasthenic crisis, classified as Myasthenia Gravis Foundation of America (MGFA) class 5, with a Myasthenia Gravis Activities of Daily Living (MG-ADL) score of 23.

The patient’s condition mandated immediate intervention, and she had a gastric tube inserted for enteral nutrition. Corticosteroids were contraindicated since she had previously experienced severe sleep disturbance and developed advanced osteoporosis after steroid use. PLEX was initiated; however, the patient’s condition deteriorated, and she was admitted to the intensive care unit (ICU). Here, she was intubated and received a further 5 PLEX treatments which did not result in any improvement of her condition. Whilst in the ICU, she became bradycardic and subsequently went into cardiac arrest, most likely induced by physostigmine use.

**Figure 1:**
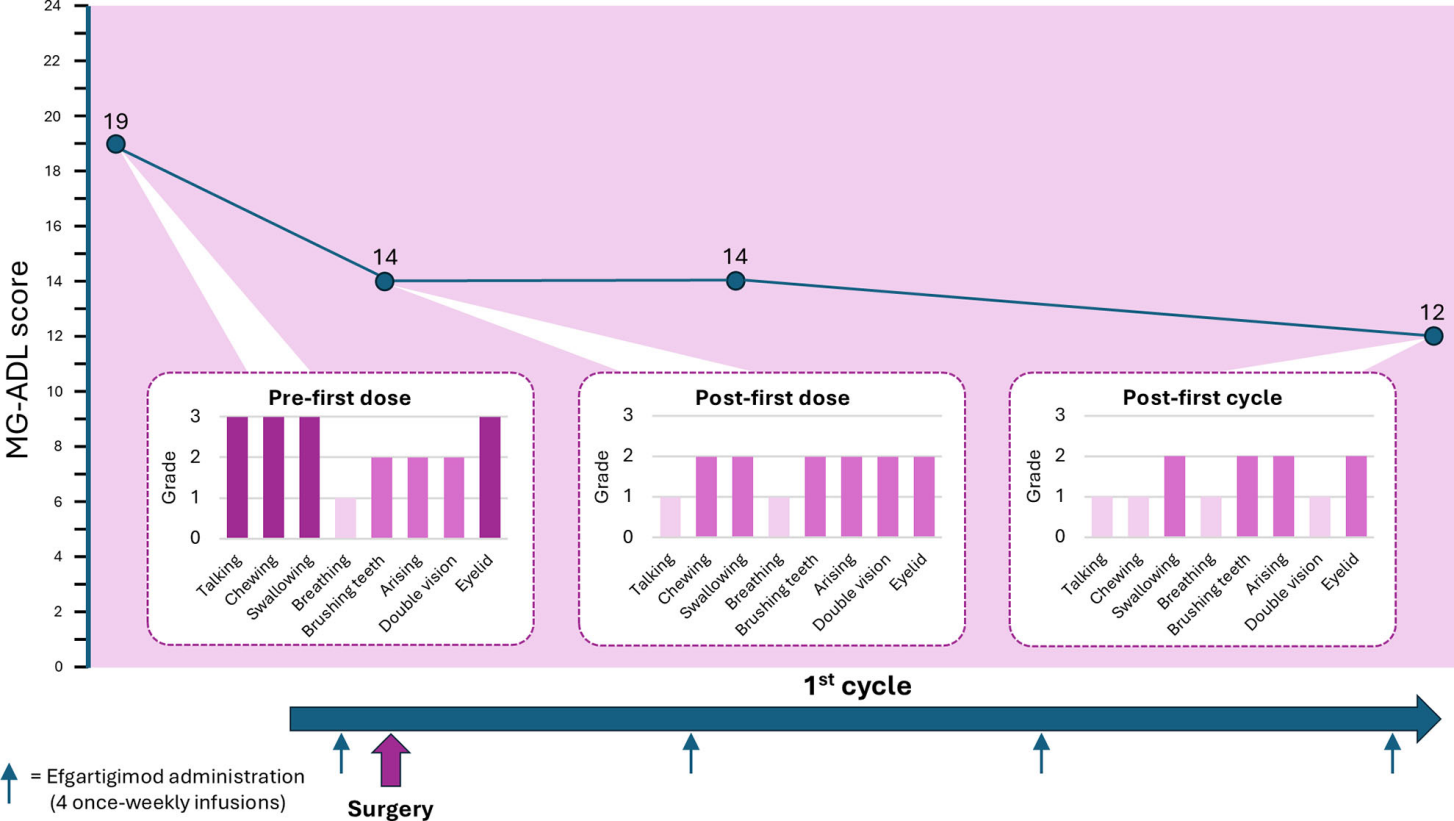
MG-ADL total- and item-specific-scores following efgartigimod treatment during the perioperative period. Timeline showing the administration of efgartigimod and associated reduction in MG-ADL score from MG-ADL 19 pre-first dose and preoperatively, to MG-ADL 12 by the end of the first cycle of efgartigimod. Item-specific scores are shown for the MG-ADL scores measured pre-first dose, post-first dose and post-first cycle of efgartigimod.

**Figure 2:**
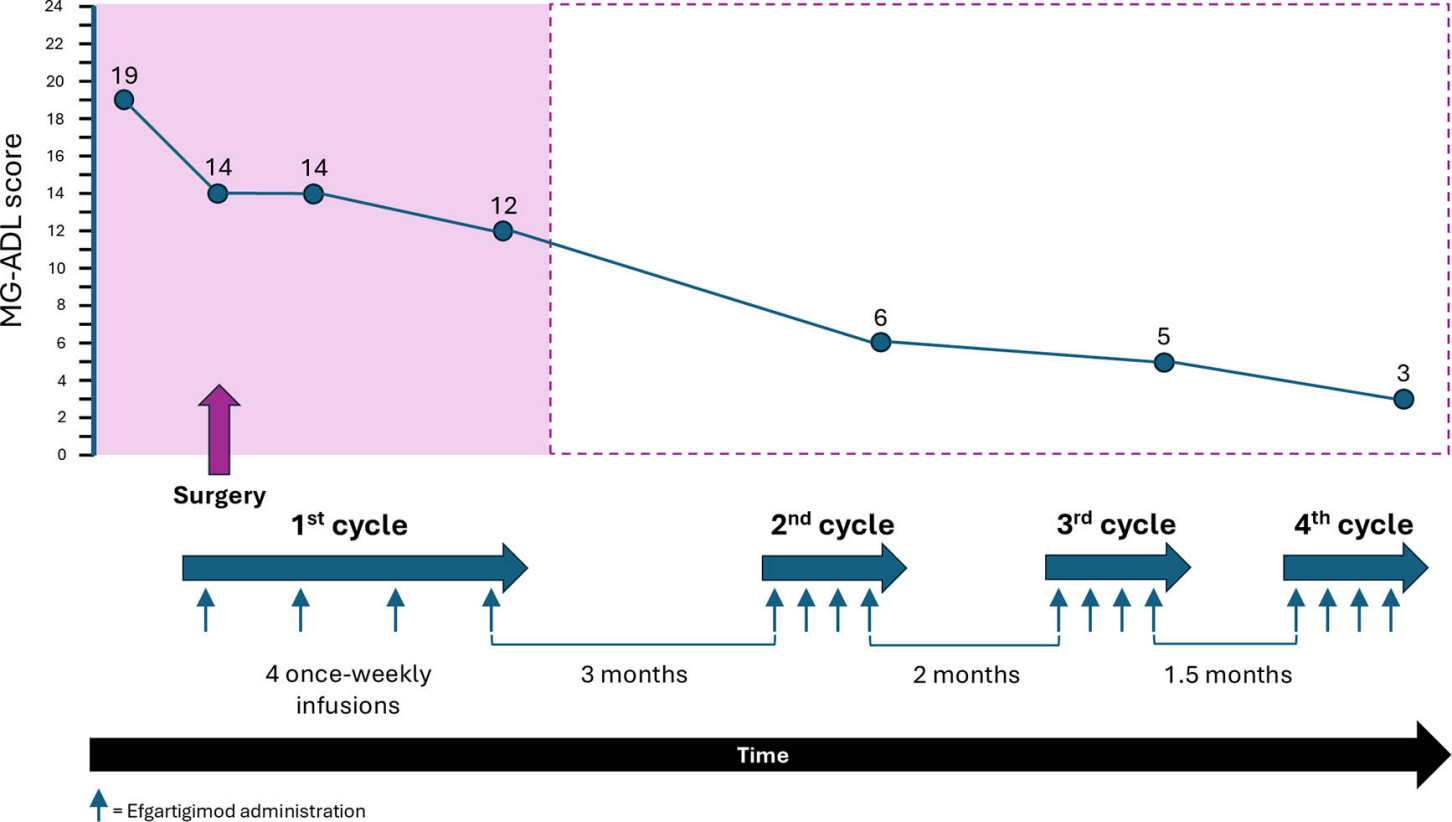
MG-ADL score following 4 cycles of efgartigimod treatment over approximately 10 months. Timeline showing the administration of efgartigimod cycles and associated reduction in MG-ADL score during the perioperative period (in pink) and over the following 9 months. The timeline is not to scale.

Whilst the patient was in ICU, the family disclosed that an echogenic finding had been discovered on bladder ultrasound prior to her hospitalization, on the chance that it may be relevant to the case. Considering this information, and the pattern and relatively rapid onset of MG symptoms in this previously healthy, though elderly, patient, suspicion was raised of active paraneoplastic disease. She underwent a whole-body CT that revealed a non-muscle-invasive bladder mass. Fourteen days after she was admitted to the ICU, she was extubated and transferred to the neurology ward in preparation for surgery to remove the bladder mass. However, her MG symptoms were not under control (MG-ADL 21), and she was still bedridden and required the use of a gastric tube. The paraneoplastic phenomena which were rendering her MG intractable made surgery an urgent need, and a decision was made to administer efgartigimod 10 mg/kg intravenously. Before the first dose her MG-ADL score was 19, which improved to MG-ADL 14 post-infusion. The patient underwent successful surgical removal of the bladder tumor 1 day after the first dose of efgartigimod. A second dose of efgartigimod was administered 5 days postoperatively, which sustained her MG-ADL score at 14. She completed a full cycle of efgartigimod treatment (a total of 4 infusions 1 week apart), reaching MG-ADL 12 at the end of the cycle (**Figure 1**). The patient experienced ankle edema with the first dose of efgartigimod, for which she was investigated. Cardiac echocardiogram, renal ultrasound, creatinine levels, ultrasound and radiography of the lower limbs did not reveal any significant findings. The edema resolved after 1 month and did not recur with further efgartigimod treatment. The patient’s MG-ADL score continued to improve over 3 more cycles of efgartigimod, reaching MG-ADL 3 after approximately 10 months (**Figure 2**).

## 2 Discussion

This case demonstrates successful response to efgartigimod in 2 complex parallel scenarios that, to my knowledge, have not previously been studied: first, in the treatment of a patient in myasthenic crisis secondary to active paraneoplastic disease, and second, in the perioperative setting.

In patients with MG, the stress response induced by surgery may disrupt the delicate balance between neuromuscular transmission and muscle function, potentially worsening muscle weakness and fatigue (8, 9). The increased metabolic demands and catabolic effects of surgery may further strain weakened muscles and contribute to postoperative complications (10). Additionally, the use of anesthesia and neuromuscular blocking agents during surgery can complicate the function of the neuromuscular junction, requiring careful management to avoid excessive muscle weakness (4, 6, 11). Anesthesia-related factors, including drug selection and dosages, must be tailored to minimize the risk of myasthenic crises (6). The perioperative period thus demands close monitoring and individualized care to navigate the physiological stress of surgery and mitigate the potential worsening of MG symptoms (4, 6).

The current patient was complex to manage, owing to her elderly and frail condition. Her paraneoplastic disease was severe enough to render her in myasthenic crisis, and intubation was extremely difficult. The priority was to stabilize the patient’s condition before undertaking surgery. Preoperative assessment of patients with MG must involve close collaboration with the patient’s neurologist to ensure optimal stabilization and guide postoperative care (6). Given the limited reliability of serological markers, clinical evaluation remains the best determinant of a patient’s optimized condition (6). As with thymectomy, achieving optimal control before elective surgery is critical, even if it necessitates delaying the procedure (6). Ideally, patients should reach a stable MGFA Minimal Manifestation Status or better, although this may not always be achievable (6).

In this case, the patient was not responsive to repeated PLEX therapy, and IVIg, which is contraindicated in hypercoagulable states, was ruled out due to her cardiac comorbidity and neoplastic disease (7). Additionally, the limited availability of IVIg in Israel often prevents its use. The decision to administer off-label efgartigimod was made based on a lack of other available treatment options, the urgent need to optimize the patient’s condition before surgery, and efgartigimod’s known rapid onset of action (12). Efgartigimod is a neonatal Fc receptor blocker with established efficacy in the treatment of generalized MG in adult patients who are anti-AChR antibody positive, though it has not been studied in patients with MGFA class 5 (12–15). This observation highlights an essential clinical point. PLEX works by removing circulating autoantibodies, while efgartigimod, as a neonatal Fc receptor antagonist, accelerates the degradation of IgG autoantibodies. It is possible that the pathogenic autoantibody load was exceptionally high or that the autoantibody dynamics in this patient were not effectively addressed by PLEX alone. The different mechanisms of action likely explain the observed discrepancy in therapeutic responses. Furthermore, the rapid and sustained response to efgartigimod in this critically ill patient underscores its potential utility in severe, refractory MG cases, particularly when other treatment modalities are limited by clinical contraindications or resource availability.

Efgartigimod likely facilitated rapid stabilization before surgery, while tumor removal may have further reduced the underlying paraneoplastic immune response. The patient’s condition improved quickly after the first dose of efgartigimod, enabling successful tumor resection without any deterioration during or after surgery. Efgartigimod administration and surgical intervention significantly improved the patient’s respiratory function, swallowing, and speech clarity. These improvements corresponded with a notable reduction in her MG-ADL score, reflecting sustained functional recovery. Her MG-ADL score continued to improve steadily throughout the postoperative period and the following months.

## 3 Conclusion

This case provides important experience of efgartigimod use in a complex patient, resulting in favorable outcomes and acceptable safety. Multiple challenges were at play, including the need for preoperative management of a hemodynamically unstable and frail patient in myasthenic crisis. Efgartigimod may provide a novel treatment strategy for stabilizing patients before surgery and enhancing postoperative recovery, adding a valuable dimension to the treatment of acute crises in autoimmune and paraneoplastic disorders. Further study of the use of efgartigimod in myasthenic crisis, and in the perioperative management of patients with MG, is warranted.

## Data Availability

The raw data supporting the conclusions of this article will be made available by the authors, without undue reservation.
